# Efficacy and safety of chemoradiation therapy using one-shot cisplatin via hepatic arterial infusion for advanced hepatocellular carcinoma with major macrovascular invasion: a single-arm retrospective cohort study

**DOI:** 10.1186/s12876-022-02359-x

**Published:** 2022-06-02

**Authors:** Kensuke Naruto, Tomokazu Kawaoka, Kenichiro Kodama, Yutaro Ogawa, Kei Amioka, Yuki Yoshikawa, Chihiro Kikukawa, Yousuke Suehiro, Kenji Yamaoka, Yuwa Ando, Yumi Kosaka, Shinsuke Uchikawa, Takashi Nakahara, Eisuke Murakami, Atsushi Ono, Takuro Uchida, Masami Yamauchi, Wataru Okamoto, Shoichi Takahashi, Michio Imamura, Keigo Chosa, Kazuo Awai, Katsumaro Kubo, Yasushi Nagata, Kazuaki Chayama, Hiroshi Aikata

**Affiliations:** 1 Department of Gastroenterology and Metabolism, Hiroshima University, 1-2-3, Kasumi, Minami-ku, Hiroshima, 734-8551 Japan; 2 Diagnostic Radiology, Hiroshima University, 1-2-3, Kasumi, Minami-ku, Hiroshima, 734-8551 Japan; 3 Therapeutic Radiology, Institute and Graduate School of Biomedical Sciences, Hiroshima University, 1-2-3, Kasumi, Minami-ku, Hiroshima, 734-8551 Japan; 4 Research Center for Hepatology and Gastroenterology, Hiroshima University, 1-2-3, Kasumi, Minami-ku, Hiroshima, 734-8551 Japan; 5 Institute of Physical and Chemical Research (RIKEN) Center for Integrative Medical Sciences, 1-7-22, Suehiro, Tsurumi-ku, Yokohama, 230-0045 Japan; 6 Department of Medicine and Molecular Science, Division of Frontier Medical Science, Programs for Biomedical Research, Graduate School of Biomedical Sciences, Hiroshima University, 1-2-3, Kasumi, Minami-ku, Hiroshima, 734-8551 Japan

**Keywords:** Hepatocellular carcinoma, Macrovascular invasion, Hepatic arterial infusion chemotherapy, Radiation therapy

## Abstract

**Background:**

Patients with hepatocellular carcinoma (HCC) and macrovascular invasion (MVI) who receive systemic chemotherapy have a poor prognosis. This study aimed to determine if one-shot cisplatin (CDDP) chemotherapy via hepatic arterial infusion (HAI) combined with radiation therapy (RT) prior to systemic chemotherapy could improve the outcomes of these patients.

**Methods:**

This study consisted of 32 HCC patients with the following eligibility criteria: (i) portal vein invasion 3/4 and/or hepatic vein invasion 2/3; (ii) received one-shot CDDP via HAI; (iii) received RT for MVI, (iv) a Child–Pugh score ≤ 7; and (v) an Eastern Clinical Oncology Group Performance Status score of 0 or 1. To determine the therapeutic effect, we collected information on patient characteristics and took contrast-enhanced computed tomography at the start of the therapy and every 2 to 4 months after the start of therapy. We evaluated the overall response of the tumor and tumor thrombosis according to modified Response Evaluation Criteria in Solid Tumors. We assessed patient data using the Mann–Whitney U and Fisher exact tests and evaluated overall survival and progression-free survival using the log-rank test.

**Results:**

The overall response rate at the first evaluation performed a median of 1.4 weeks after HAI was 16% for the main intrahepatic tumor and 59% for the MVI. The best responses were the same as those of the first-time responses. The duration of median survival was 8.6 months, and progression-free survival of the main intrahepatic tumor was 3.2 months. Predictive factors for overall survival were the relative tumor volume in the liver and the first therapeutic response of MVI. There were no severe adverse events or radiation-induced hepatic complications.

**Conclusions:**

One-shot CDDP via HAI and RT were well tolerated and showed immediate and favorable control of MVI. Thus, this combination shows potential as a bridging therapy to systemic chemotherapy.

## Introduction

In global cancer statistics, hepatocellular carcinoma (HCC) is a leading cause of cancer-related death [1]. Furthermore, the prognosis of HCC patients with macrovascular invasion (MVI) is poor [2]. HCC frequently occurs in patients with chronic hepatitis or liver cirrhosis due to hepatitis B or C viral infection, alcohol use, nonalcoholic steatohepatitis, or diabetes [3]. The survival of patients with HCC has improved with the development of diagnostic modalities and progress in treatment options, such as radical surgical resection, radiofrequency ablation, microwave ablation, percutaneous ethanol injection [4, 5], and transcatheter arterial chemoembolization [6, 7], radiation therapy (RT) [8], chemotherapy via hepatic arterial infusion (HAI), and multiple molecular targeting agents including immune checkpoint inhibitors [9, 10]. However, the prognosis of patients with advanced HCC, especially those with advanced MVI, remains poor [11–21].

Systemic chemotherapy, such as the combination of atezolizumab plus bevacizumab (atezo + beva), is effective for patients with advanced HCC [10, 22, 23] and for patients with HCC with portal vein invasion (Vp), but the overall survival (OS) of patients with Vp was less than that of patients without Vp [23–25]. The poor prognosis of patients with Vp is a result of major MVI that causes deterioration in the preserved hepatic function and portal hypertension, and interferes with the administration of chemotherapy [10]. However, atezo + beva take 2.8 months to obtain a response, and 20% of the recipients of these agents are non-responders [23–25].

Thus, we aimed for a better outcome for patients with advanced HCC with the use of one-shot cisplatin (CDDP) via HAI plus RT to obtain rapid control of MVI before the administration of systemic chemotherapy. HAI, used for either continual infusion via an implanted reservoir system or to administer one-shot CDDP, remains insufficient by itself to control MVI. Therefore, we attempted to examine the effects of combining one-shot CDDP via HAI with RT for patients with MVI.

Previously we reported the effects of treating HCC patients by combining CDDP administered via an implanted reservoir system with RT [26–31]; however, there were complications with the implanted reservoir system. Moreover, only a few studies are available that address one-shot CDDP via HAI combined with RT. Therefore, this study aimed to evaluate the safety and efficacy of one-shot CDDP via HAI combined with RT for patients before systemic chemotherapy.

## Materials and methods

### Patients

In this single-arm retrospective cohort study, patients with HCC were treated with one-shot CDDP via HAI plus RT for severe MVI. Severe MVI contains the tumor thrombus in the hemilobar branch of the portal vein (Vp3), the tumor thrombus in the main trunk or bilobar branches of the portal vein (Vp4), tumor thrombus in the main trunk of left hepatic vein, middle hepatic vein, or right hepatic vein (Vv2), and the tumor thrombus in inferior vena cava (Vv3).

The patients who met the following criteria were enrolled: (i) presence of Vp3–4 or Vv2–3, (ii) Child–Pugh score of 5–7, (iii) Eastern Clinical Oncology Group Performance Status score of 0–1, (iv) no history of systemic therapy, and (v) HAI performed from September 2004 to September 2020. This study was approved by the Institutional Review Board of Hiroshima University Hospital, and was based on the ethical principles of the Declaration of Helsinki 7th version in 2013 (project identification code number: E-968). Detailed written informed consent was obtained from each patient for each treatment.

### Therapeutic protocol

#### HAI

In Japan, chemotherapy via HAI with single bolus injection of CDDP (one-shot CDDP) is a common treatment option for HCC patients with MVI. The effectiveness of one-shot HAI has been previously reported [30]. The patients were administered CDDP at a dose of 65 mg/m^2^ (maximum dose, 100 mg/m^2^) at a rate of 2 mL/min through a percutaneous catheter into the entire liver. The dose was decreased by 75% if the patient’s estimated glomerular filtration rate was < 60 mg/min/1.73 m^2^. When CDDP was infused into a part of the liver, the dose was adjusted roughly by the proportion of the tumor volume in the liver, which was determined via computed tomography (CT) images. Adequate hydration was also ensured both before and after the administration of CDDP to prevent CDDP-induced renal dysfunction.

#### RT for MVI

Every study patient was administered three-dimensional conformal radiation therapy (3D-CRT) concomitantly with one-shot CDDP via HAI. The treatments were administered in the Division of Radiation Oncology at our hospital [21]. The treatment protocol was as follows: 3D–CRT was performed with 18, 10, or 6 MV high-energy photon beams that were delivered by a linear accelerator (CLINAC 2300 C/D or CLINAC iX linear accelerator; Varian Medical Systems Inc., Palo Alto, CA, USA) using the 3D conformal technique. Planning CT was used to determine the total volume of the tumor involved in the MVI. The clinical target volume was established as the gross tumor volume plus the intrahepatic tumor volume in the basal portion of the MVI. The planning target volume comprised the clinical target volume plus a 10–20-mm margin in all directions to account for internal motion and set-up errors. Data consisting of the outlined target volume; total volume of liver tissue; and volume of at-risk structures, including the spinal cord, both kidneys, and nearby targets of the intestinal tract; were transferred to the treatment planning system (Pinnacle 3; Philips Medical Systems, Eindhoven, the Netherlands) with reference to the diagnostic contrast-enhanced CT images. The irradiation dose was 39 Gy depending on the volume of normal tissue and liver function, with 95% of the planning target volume receiving at least 95% of the irradiation dose.

### Assessment of treatment efficacy

The therapeutic effectiveness was evaluated according to changes in tumor volume. The response to treatment was assessed by contrast-enhanced CT. The baseline CT image was taken before HAI. The image for the first-time evaluation was taken after the completion of RT for MVI and then every 2–3 months for follow-up. Especially in the case of hypervascular tumors such as HCC, a decrease in tumor volume during the arterial phase, as determined by contrast-enhanced CT, often indicates a decrease in tumor activity prior to tumor necrosis. Thus, response was determined according to the modified Response Evaluation Criteria in Solid Tumors (mRECIST) [32]. According to mRECIST, the therapeutic effect of the treatment on MVI was determined by measuring the longest diameter and the degree of contrast of the MVI.1. We also evaluated the response based on RECIST 1.1.

### Evaluation of treatment-related adverse events

Assessment of safety included documentation of adverse drug reactions at each physical examination, measurement of vital signs, and examination of clinical laboratory data. Adverse drug reactions were defined according to the National Cancer Institute Common Terminology Criteria for Adverse Events (NCI-CTCAE) v5.0 (http://ctep.cancer.gov/protocolDevelopment/electronic_applications/docs/ctcaev3.pdf).

Radiation-induced liver disease (RILD) was divided into “classical” and “non-classical” RILD [33]. The endpoint of non-classical RILD was described in patients with poor liver function. Classical RILD, which usually occurs between 2 weeks and 3 months after irradiation, involves anicteric hepatomegaly and ascites, and elevation of the alkaline phosphatase level to at least twofold the upper limit of normal or the pretreatment value in the absence of tumor progression. Classical RILD can occur in patients with good liver function. Non-classical RILD, which usually occurs between 1 week and 3 months after irradiation, involves elevation of the alkaline phosphatase level to greater than fivefold the upper limit of normal. Furthermore, it involves CTCAE grade 4 in patients with a baseline value greater than fivefold the upper limit of normal within 3 months after the completion of RT, or a decrease in liver function (defined by an increase in the Child–Pugh score of > 2 points), in the absence of classical RILD.

### Statistical analysis

The Mann–Whitney U test and the Fisher exact test were used for statistical analysis of patient characteristics. Overall survival (OS) was calculated based on the initial date of therapy. Progression-free survival (PFS) was calculated based on the dates of the initial therapy and the diagnosis of progressive disease (PD). These parameters were plotted and assessed by the Kaplan–Meier life-table method, and differences in survival between subgroups were evaluated by the log-rank test. Multivariate analysis of predictors of OS was assessed by binomial logistic regression with backward elimination according to the *p* value. *P* < 0.10 was considered to demonstrate a tendency, whereas *p* < 0.05 was considered to be statistically significant. EZR (Saitama Medical Center, Jichi Medical University, Saitama, Japan), which is a graphical user interface for R (The R Foundation for Statistical Computing, Vienna, Austria) was used for statistical analysis. EZR is a modified version of R Commander that was created to add statistical functions that are frequently used in biostatistics [34].

## Results

### Patient clinical characteristics at the start of HAI

A total of 32 patients were enrolled in this retrospective cohort study. The characteristics of the patients and clinical data at the time of initial treatment are summarized in Table 1. The median age of the patients was 69.5 years (range, 40–85 years), and 28 patients were male. Child–Pugh scores of 5, 6, and 7 were noted in 9 (28%), 10 (31%), and 13 (41%) patients, respectively. Regarding the etiology of HCC, 9 and 10 patients were positive for hepatitis B surface antigen and hepatitis C virus antibodies, respectively; and 1 patient was positive for both viruses. The relative tumor volume in the liver was < 50% in 19 (59%) patients. The median liver tumor size was 103 mm (range, 36–185 mm). The number of patients with HCC with Vp4 was 15 (47%), and those with Vp3 was 13 (41%). A total of 10 patients (31%) had extrahepatic metastases. The median alpha-fetoprotein (AFP) level was 713.2 ng/mL (range, 1.3–3,686,000 ng/mL). The median des-γ-carboxy prothrombin level was 10,039 mAU/mL (range, 36–327,600 ng/mL). A total of 23 patients received additional systemic therapy (median 1.5 month after HAI); 6 received sorafenib, and 13 received lenvatinib, and 4 received investigational agents such as pembrolizumab and the combination of durvalumab and tremelimumab. Four of 13 patients with Child–Pugh score 7 points (30%) had met the conditions for use of systemic chemotherapy.

**Table 1:** Clinical characteristics of patients who received one-shot CDDP via HAI plus RT

Characteristics	Median (range) or number of patients
Age (years)	69.5 (40–85)
Gender (male/female)	28/4
ECOG-Performance Status (0/1)	25/7
Etiology (HBV/HCV/HBV + HCV/other)	9/10/1/12
Total bilirubin (mg/dL)	0.9 (0.4–1.5)
Albumin (g/dL)	3.5 (2.6–4.6)
Prothrombin consumption test (%)	86.0 (6.2–111)
Child–Pugh score (5/6/7)	9/10/13
ALBI score	− 2.17 (− 3.24– − 1.28)
mALBI grade (1/2a/2b/3)	5/7/19/1
Size of liver tumor (mm)	103.2 (36–185)
Number of intrahepatic tumors (< 4/ ≥ 4)	18/14
Relative tumor volume in the liver (< 50%/ ≥ 50%)	19/13
Vp (3/4)	13/15
Vv (0/1/2/3)	27/1/1/3
Extrahepatic spread (without/with)	22/10
HCC stage (III/IVa/IVb)^a^	4/6/22
BCLC stage (A/B/C)	0/0/32
Alpha-fetoprotein (ng/mL)	713.2 (1.3–3,686,000)
Des-γ-carboxy prothrombin (mAU/mL)	10,039 (36–327,600)
Additional systemic therapy (with/without)	23/9

The clinical characteristics of the HCC patients with MVI who received one-shot CDDP chemotherapy via HAI plus RT. *HCC* hepatocellular carcinoma; *MVI* macrovascular invasion; *HAI* hepatic arterial infusion; *CDDP* cisplatin; *HAI* hepatic arterial infusion; *RT* radiation therapy; *ECOG-Performance Status* Eastern Cooperative Oncology Group Performance Status; *HBV* hepatitis B virus; *HCC* hepatocellular carcinoma; *HCV* hepatitis C virus; ALBI score = log_10_([total-bilirubin(mg/dL)]*17.1*0.66 − [albumin(g/dL)]*10*0.085; mALBI grade 1: 2a: 2b: 3 = ALBI score ≤  − 2.6: >  − 2.6 to <  − 2.27: ≥  − 2.27 to ≤  − 1.39: >  − 1.39; *Vp* portal vein invasion; *Vv* venous invasion; *BCLC stage* Barcelona Clinic liver cancer stage

^a^According to the Liver Cancer Study Group of Japan

### Overall response rate to HAI and RT

Based on mRECIST criteria, which evaluates changes in volume as determined by contrast-enhanced CT, the best overall response rate (ORR) was 16%. The best ORRs of tumor involved in MVI and the main tumor were 59% and 16%, respectively. The disease control rates (DCR) of tumor involved in MVI and the main tumor were 97% and 41%, respectively. The complete response (CR), partial response (PR), stable disease (SD), and progressive disease (PD) rates of the first-time response assessment (median 1.4 month) of the MVI were 0%, 59%, 28%, and 3%, respectively; and those of the main tumor were 0%, 16%, 25%, and 59%, respectively (Table 2a).

**Table 2:** a. Responses of tumor in MVI and main tumor to one-shot CDDP chemotherapy plus RT, as assessed by mRECIST. b Responses of tumor in MVI and main tumor to one-shot CDDP chemotherapy plus RT, as assessed by RECIST 1.1

	HAI + RT (n = 32)					
	First-time response (median, 1.4 month)			Best response		
	Whole tumor	Main tumor	MVI	Whole tumor	Main tumor	MVI
(**a**)						
CR	0	0	0	0	0	0
PR	16 (5)	16 (5)	59 (19)	16 (5)	16 (5)	59 (19)
SD	25 (8)	25 (8)	28 (12)	25 (8)	25 (8)	28 (12)
PD	59 (19)	59 (19)	3 (1)	59 (19)	59 (19)	3 (1)
ORR	16 (5)	16 (5)	59 (19)	16 (5)	16 (5)	59 (19)
DCR	41 (13)	41 (13)	97 (31)	41 (13)	41 (13)	97 (31)
(**b**)						
CR	0	0	0	0	0	0
PR	9 (3)	9 (3)	25 (8)	16 (5)	16 (5)	56 (18)
SD	32 (10)	32 (10)	72 (23)	25 (8)	25 (8)	43 (13)
PD	59 (19)	59 (19)	3 (1)	59 (19)	59 (19)	3 (1)
ORR	9 (3)	9 (3)	25 (8)	16 (5)	16 (5)	56 (18)
DCR	41 (13)	41 (13)	97 (31)	41 (13)	41 (13)	97 (31)

The best and first-time response of tumor in MVI and main tumor to one shot CDDP chemotherapy via HAI plus RT, as assessed by mRECIST and RECIST 1.1. The median period of the first-time response was assessed was 1.4 months from HAI. Data are presented as percentages (numbers). *MVI* macrovascular invasion; *CDDP* cisplatin; *RT* radiation therapy; *CR* complete response; *PR* partial response; *SD* stable disease; *PD* progressive disease; *ORR* overall response rate; *DCR* disease control rate; *mRECIST* modified Response Evaluation Criteria in Solid Tumors

The response based on RECIST v1.1 of the first-time response assessment of the MVI were 0%, 25%, 72%, and 3%, respectively; and those of the main tumor were 0%, 9%, 32%, and 59%, respectively. The best response of the MVI were 0%, 56%, 43%, and 3%, respectively; and those of the main tumor were 0%, 16%, 25%, and 59%, respectively. (Table 2b).

### OS and factors affecting OS

**Fig. 1:**
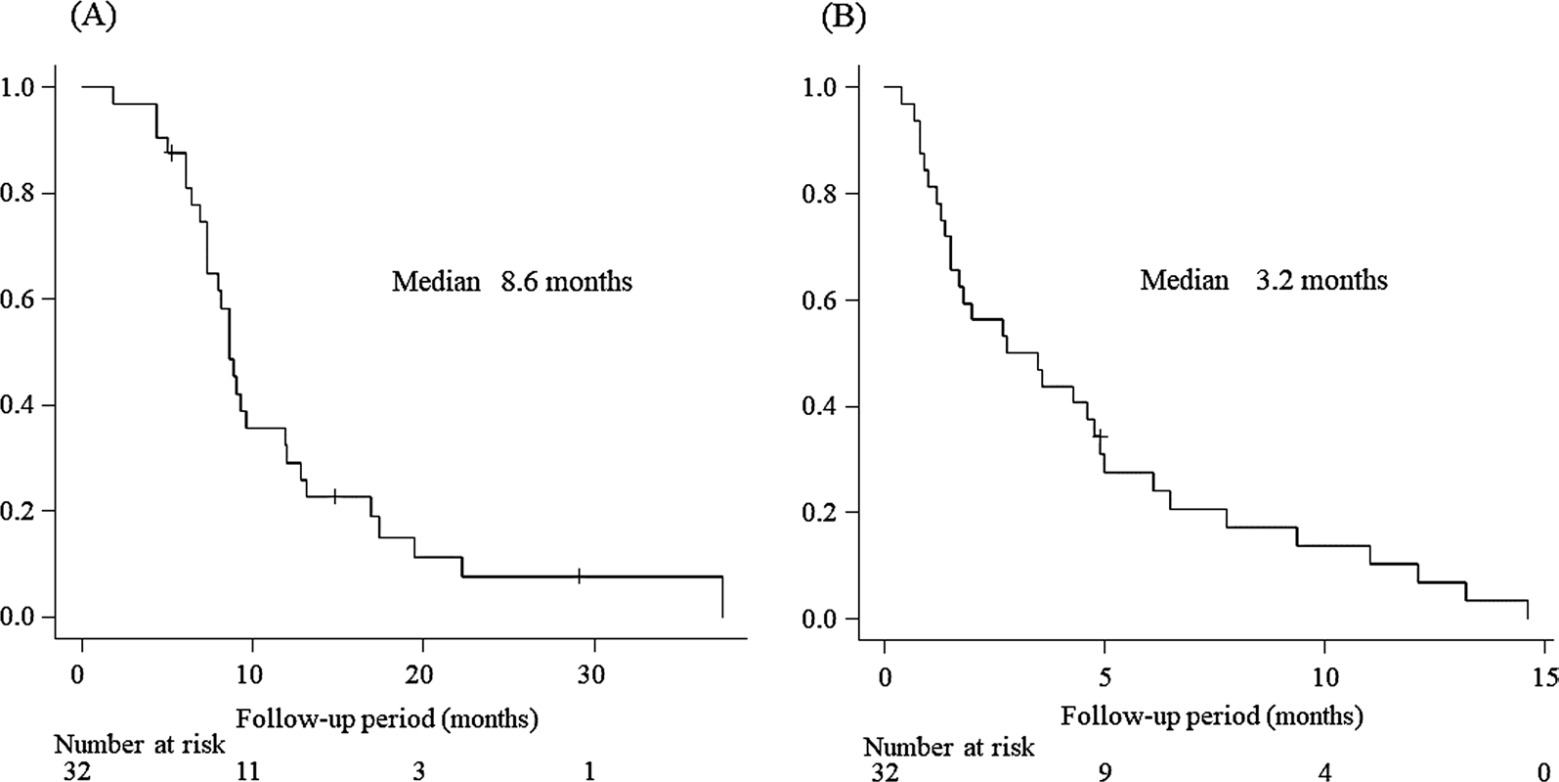
**A** Overall survival of patients with HCC treated with the combination of one-shot CDDP via HAI and RT. **B** Progression-free survival of patients with HCC treated with the combination of one-shot CDDP via HAI and RT. *HCC* hepatic cell carcinoma; *CDDP* cisplatin; *HAI* hepatic arterial infusion; *RT* radiation therapy

The median OS time of the study patients was 8.6 months (Fig. 1A). Univariate analysis identified the following as significant factors predicting unfavorable OS: relative tumor volume in the liver ≥ 50% (*p* = 0.013), AFP ≥ 400 ng/mL (*p* = 0.048), and SD as the first-time response of tumor in the MVI (*p* = 0.002). Extrahepatic spread (*p* = 0.077) showed a tendency for unfavorable OS.

**Fig. 2:**
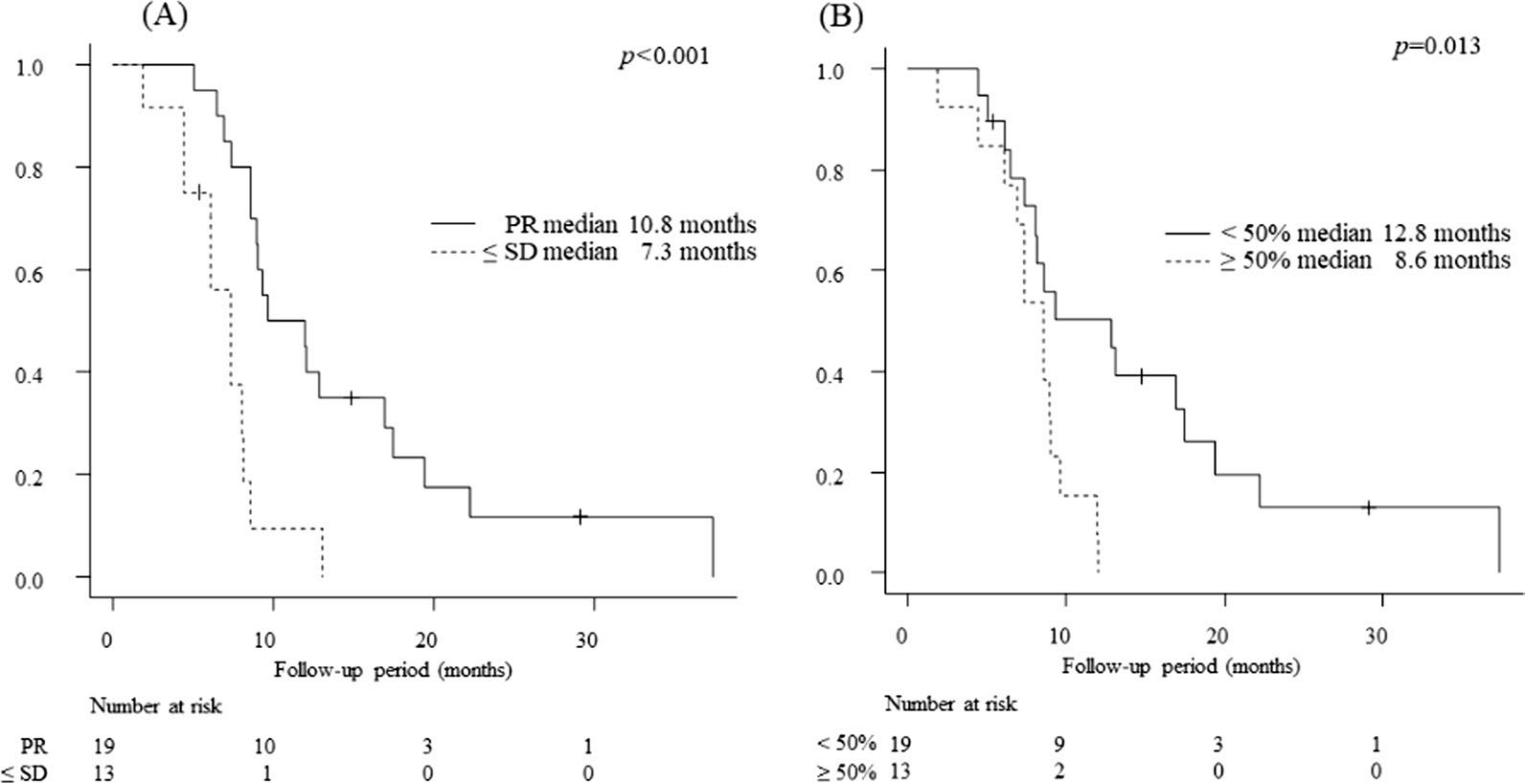
**A** Overall survival of patients with HCC treated with the combination of one-shot CDDP chemotherapy via HAI and RT, divided by the first-time response of tumor thrombus in the portal vein PR/SD. **B** Overall survival of patients with HCC treated with the combination of one-shot CDDP chemotherapy via HAI and RT, divided by the relative tumor volume < 50%/ ≥ 50%. *HCC* hepatocellular carcinoma; *CDDP* cisplatin; *HAI* hepatic arterial infusion; *RT* radiation therapy; *PR* partial response; *SD* stable disease

By multivariate analysis, relative tumor volume in the liver ≥ 50% (odds ratio [OR], 4.501; 95% confidence interval [CI], 1.61–12.59; *p* = 0.041) and the first-time response of tumor in the MVI as PD or SD (OR, 7.396; 95% CI, 2.639–20.72; *p* < 0.001) remained significant and independent factors for predicting poor OS (Fig. 2A, B; and Table 3).

**Table 3:** Risk factors of overall survival in HCC patients with MVI who received one-shot CDDP plus RT

Parameter	Univariate analysis	Multivariate analysis		
	*p* value	OR	95% CI	*p* value
Age (≥ 65/ < 65 years)	0.303			
Gender (female/male)	0.599			
Etiology (with viral hepatitis/without viral hepatitis)	0.528			
ECOG- Performance Status (0/ ≥ 1)	0.924			
Albumin (≥ 3.5/ < 3.5 g/dL)	0.692			
Prothrombin consumption test (≥ 70%/ < 70%)	0.789			
Child–Pugh score (6 ≥ /5)	0.619			
mALBI grade (≥ 2b/ ≤ 2a)	0.129			
Diameter of main tumor (≥ 70/ < 70 mm)	0.11			
Number of tumors in the liver (≥ 4/ < 4)	0.478			
Relative tumor volume in the liver (≥ 50%/ < 50%)	0.011	4.501	1.61–12.59	0.041
Vp (3/4)	0.801			
Vv (with/without)	0.539			
Extrahepatic spread (with/without)	0.077			0.367
Alpha-fetoprotein (≥ 400/ < 400 ng/mL)	0.048			0.403
Des-γ-carboxy prothrombin (≥ 400/ < 400 mAU/mL)	0.694			
First response of MVI (SD or PD/ CR or PR)	0.002	7.396	2.639–20.72	< 0.001

Univariate and multivariate analysis of risk factor of overall survival in HCC patients with MVI who received one-shot CDDP chemotherapy via HAI plus RT. *HCC* hepatocellular carcinoma; *MVI* macrovascular invasion; *CDDP* cisplatin; *RT* radiation therapy; *OR* odds ratio; *CI* confidence interval; *ECOG-Performance Status* Eastern Cooperative Oncology Group Performance Status; ALBI score = log_10_([total-bilirubin(mg/dL)]*17.1*0.66 − [albumin(g/dL)]*10*0.085; mALBI grade 1: 2a: 2b: 3 = ALBI score ≤  − 2.6: >  − 2.6 to <  − 2.27: ≥  − 2.27 to ≤  − 1.39: >  − 1.39; *Vp* portal vein invasion; *Vv* venous invasion; *SD* stable disease; *PD* progressive disease; *CR* complete response; *PR* partial response; *HAI* hepatic arterial infusion

### PFS and factors affecting PFS

The median PFS time was 3.2 months (Fig. 1B). Univariate analysis identified relative tumor volume in the liver of ≥ 50% (*p* < 0.001) as a significant predictor of poor PFS. By multivariate analysis, relative tumor volume in the liver of ≥ 50% (OR, 2.79; 95% CI, 1.247–6.243; *p* = 0.013) remained a significant and independent factor for predicting unfavorable PFS (Table 4).

**Table 4:** Risk factors predicting progression-free survival in HCC patients with MVI who received one-shot CDDP plus RT

Parameter	Univariate analysis	Multivariate analysis		
	*p* value	OR	95% CI	*p* value
Age (≥ 65/ < 65 years)	0.753			
Gender (female/male)	0.269			
Etiology (with viral hepatitis/without viral hepatitis)	0.991			
ECOG-PS (0/ ≥ 1)	0.799			
Albumin (≥ 3.5/ < 3.5 g/dL)	0.838			
Prothrombin consumption test (≥ 70%/ < 70%)	0.619			
Child–Pugh score (≥ 6/5)	0.375			
mALBI grade (≥ 2b/ ≤ 2a)	0.505			
Diameter of main tumor (≥ 70/ < 70 mm)	0.479			
Number of tumors in the liver (≥ 4/ < 4)	0.201			
Relative tumor volume in the liver (≥ 50%/ < 50%)	0.009	2.79	1.247–6.243	0.013
Vp (3/4)	0.949			
Vv (with/without)	0.713			
Extrahepatic spread (with/without)	0.304			
Alpha-fetoprotein (≥ 400/ < 400 ng/mL)	0.135			
Des-γ-carboxy prothrombin (≥ 400/ < 400 mAU/mL)	0.906			
Best response of MVI (CR or PR/SD or PD)	0.132			

Univariate and multivariate analysis of risk factor of overall survival in HCC patients with MVI who received one-shot CDDP chemotherapy via HAI plus RT. *HCC* hepatocellular carcinoma; *MVI* macrovascular invasion; *CDDP* cisplatin; *RT* radiation therapy; *OR* odds ratio; *CI* confidence interval; *ECOG-Performance Status* Eastern Cooperative Oncology Group Performance Status; ALBI score = log_10_([total-bilirubin(mg/dL)]*17.1*0.66 − [albumin(g/dL)]*10*0.085; mALBI grade 1: 2a: 2b: 3 = ALBI score ≤  − 2.6: >  − 2.6 to <  − 2.27: ≥  − 2.27 to ≤  − 1.39: >  − 1.39; *Vp* portal vein invasion; *Vv* venous invasion; *SD* stable disease; *PD* progressive disease; *CR* complete response; *PR* partial response; *HAI* hepatic arterial infusion

### Treatment-related adverse events

Treatment-related toxicities were observed in 16 of 32 patients (50%) from the time of HAI to the time of the second response evaluation (median 4.3 month). Whether AEs were present or not, we tried to maintain each patient’s condition. Common adverse events were fever (25%, 8 cases), elevated transaminase level (16%, 5 cases), anorexia (6%, 2 cases), elevated ammonia level (6%, 2 cases), ascites (3%, 1 case), and general fatigue (3%,1 case). The adverse events in this study were categorized as CTCAE v5.0 grades 1 or 2. None of the patients who received chemotherapy via HAI and RT for advanced HCC with MVI developed hepatic failure that met the criteria for classical or nonclassical RILD (Table 5).

**Table 5:** Adverse events after administration of one-shot CDDP via HAI and RT up to the time of the second response evaluation (median 4.3 month), as categorized by NCI-CTCAE v5.0

	All (N = 32)	
Adverse event	Any grade	Grade ≥ 3
Fever	25 (8)	0
Aspartate aminotransferase increased	16 (5)	0
Alanine aminotransferase increased	12 (4)	0
Anorexia	6 (2)	0
Elevated ammonia	6 (2)	0
Ascites	3 (1)	0
Fatigue	3 (1)	0

The adverse events after administration of one-shot CDDP via HAI and RT up to time of the the second response evaluation (median 4.3 month), as categorized by NCI-CTCAE v5.0. Data are presented as percentages (numbers). *CDDP* cisplatin; *HAI* hepatic arterial infusion; *RT* radiation therapy; *NCI-CTCAE* National Cancer Institute Common Terminology Criteria for Adverse Event

## Discussion

In a previous study, we reported that the combination of HAI via the reservoir system and RT for patients with HCC plus MVI led to improved outcomes [26, 29–31]. In this study, we analyzed the clinical outcomes of HCC patients with MVI treated by the combination of one-shot CDDP administered via HAI plus RT. The median OS and PFS times were 8.6 and 3.2 months, respectively. The ORR, evaluated 1.4 months after HAI, was 16% for the main tumor, and 59% for tumor in the MVI. There were no severe adverse events that resulted in discontinuation of the study for any of the patients. Nineteen of 32 patients received additional systemic therapy, which seems to have been well tolerated and resulted in immediate and favorable control of MVI. Since we did not use the implanted reservoir system in this study, there were no adverse events associated with an implanted port and catheter, as we reported in a previous study involving a reservoir system.

HCC is a leading cause of cancer-related death worldwide, and the prognosis of patients with unresectable HCC with major MVI is extremely poor [1, 2]. Systemic chemotherapy, such as atezo + beva, is effective for advanced HCC [10, 23] and for HCC with Vp. However, the prognosis remains poor, with 67% of patients alive 6 months after the initiation of treatment with atezo + beva [24, 25]. Kudo et al. reported that MVI is a factor leading to poor outcomes in patients with advanced HCC because MVI rapidly worsens flow in the portal vein and leads to liver failure and portal hypertension [10].

Many studies have reported the efficacy of HAI for treating patients with HCC with portal vein tumor thrombus (PVTT). This HAI regimen was based on 5-fuluolouracil (5-FU) and cisplatin (FP), and infused repeatedly via an implanted catheter and port called a reservoir system. The prospective SILIUS study, which compared sorafenib and HAI with low-dose FP did not show better results than those of the patients treated with sorafenib; however, the subgroup analysis of patients with HCC with Vp4 showed a better outcome than that of patients treated with sorafenib [28]. Fujino et al. reported that the combination of HAI with 5-FU based regimen and RT showed better ORR for patients with HCC with PVTT than with HAI without RT [26]. Kodama et al. compared the combination of HAI and RT for HCC patients with PVTT and sorafenib without RT and found respective median OS times of 9.9 vs. 5.3 months [29]. Kawaoka et al. compared the outcomes of patients treated with 5-FU regimen administered by HAI and one-shot CDDP administered by HAI and showed a comparison of the ORR and median OS time (9.1 vs. 8.6 months), but there was no statistically significant difference [30]. Kosaka et al. reported that the OS and PFS of 5-FU via HAI and RT for patients with HCC with Vp4 were 12.1 and 4.2 months, respectively, with 19.6% ORR for the main tumor, and 51% ORR for tumor in the MVI [31]. However, there are various problems related to the implanted reservoir system [26, 29–31]. Each type of HAI and RT therapy showed a rapid effect for MVI; on the other hand, there was insufficient overall control of the HCC, especially with large intrahepatic volumes over half of the liver or extrahepatic metastasis. Thus, we should consider the rapid initiation of systemic chemotherapy in patients with such advanced HCC.

The current first choice of systemic chemotherapy is atezo + beva [10, 23]. The median OS of this combination therapy was 19.2 months, which was longer than the median OS of 13.6 months for the sorafenib group [23]. However, subgroup analysis revealed that the prognosis of HCC patients with Vp4 remains poor; the median OS of Vp4 patients treated with atezo + beva was 7.6 months and that with sorafenib was 5.5 months [24]. In addition, the median time of response to atezo + beva was 2.8 months after initiation, and approximately 20% of patients were non-responders [25]. Therefore, atezo + beva might increase the risk to improve the MVI and reduce the liver preservation function.

Above all, we suggest that the combination of HAI and RT is important for the rapid control of MVI and as a bridging therapy to systemic chemotherapy. In particular, one-shot CDDP via HAI combined with RT should be a good therapeutic option because problems related to the reservoir system cannot occur.

This study has several limitations. It was a retrospective single-arm study with a small number of patients with a long observation period, during which the diagnosis and treatment of liver diseases has improved. This may have resulted in lead-time bias and length bias. Similarly, as we enrolled HCC patients with major MVI but good liver function, this may have led to over-selection bias. Accumulation of sample cases and further studies such as prospective, multi-arm studies, in addition to systemic reviews, should be considered. We hope that the results of our study warrant further studies.

## Conclusion

The combination of one-shot CDDP chemotherapy via HAI plus RT for patients with HCC and MVI showed rapid control of the MVI. No severe adverse events were observed in this study. Further studies should help to address the poor outcomes of HCC patients with MVI progression who only receive systemic chemotherapy.

## Data Availability

The datasets generated and analyzed during the current study are not publicly available due to lack of informed consent for public reuse of datasets but are available from the corresponding author on reasonable request for further studies.

## Abbreviations

HCC: Hepatocellular carcinoma
MVI: Macrovascular invasion
CDDP: Cisplatin
HAI: Hepatic arterial infusion
RT: Radiation therapy
atezo + beva: Atezolizumab plus bevacizumab
Vp: Portal vein invasion
OS: Overall survival
Vp3: Tumor thrombus in the hemilobar branch of the portal vein
Vp4: Tumor thrombus in the main trunk or bilobar branches of the portal vein
Vv2: Tumor thrombus in the main trunk of left hepatic vein, middle hepatic vein, or right hepatic vein
Vv3: Tumor thrombus in inferior vena cava
one-shot CDDP: Single bolus injection of CDDP
CT: Computed tomography
3D-CRT: Three-dimensional conformal radiation therapy
MV: Mega volt
mRECIST: Modified response evaluation criteria in solid tumors
NCI-CTCAE: National Cancer Institute Common Terminology Criteria for Adverse Event
RILD: Radiation-induced liver damage
PFS: Progression-free survival
PD: Progressive disease
AFP: Alpha-fetoprotein
ECOG-Performance Status: Eastern Cooperative Oncology Group Performance Status
HBV: Hepatitis B virus
HCV: Hepatitis C virus
ALBI score: Log_10_([total-bilirubin(mg/dL)]*17.1*0.66-[albumin(g/dL)]*10*0.085
mALBI grade 1: 2a: 2b: 3: ALBI score ≤ -2.6: > -2.6 to < -2.27: ≥ -2.27 to ≤ -1.39: > -1.39
Vv: Venous invasion
BCLC stage: Barcelona Clinic liver cancer stage
ORR: Overall response rates
DCR: Disease control rates
CR: Complee response
PR: Partial response
SD: Stable disease
OR: Odds ratio
CI: Confidence interval
PVTT: Portal vein tumor thrombus
5-FU: 5-Fluoluracil
FP: 5-Fluolouracil and cisplatin
